# Risk factors associated with reintubations in children undergoing foreign body removal using flexible bronchoscopy: a single-center retrospective cross-sectional study

**DOI:** 10.1186/s12871-022-01756-9

**Published:** 2022-07-13

**Authors:** Su-Jing Zhang, Hong-Bin Gu, Min Zhou, Min-Yi Lin, Long-Xin Zhang, Xiu-Ying Chen, Guo-Lin Lu

**Affiliations:** 1 Department of Anesthesiology, Fujian Maternity and Child Health Hospital, College of Clinical Medicine for Obstetrics & Gynecology and Pediatrics, Fujian Medical University, Fuzhou, China; 2 Department of Anesthesiology, College of Clinical Medicine for Obstetrics & Gynecology and Pediatrics, Fujian Medical University, Fujian Branch of Shanghai Children’s Medical Center Affiliated to Shanghai Jiaotong University School of Medicine, Fujian Children’s Hospital, Fuzhou, China; 3 Fujian Key Laboratory of Women and Children’s Critical Diseases Research, Fuzhou, China; 4 Department of anesthesia, Shanghai Children’s Medical Center, School of Medicine, Shanghai Jiao Tong University, 1678 Dongfang Road, Shanghai, China

**Keywords:** Reintubation, Foreign body, Flexible bronchoscopy

## Abstract

**Background:**

Reintubation is a severe complication during foreign body (FB) removal that uses flexible bronchoscopy.

**Objective:**

To investigate the incidence and risk factors for reintubations in children undergoing FB extraction by flexible bronchoscopy in a single center.

**Design:**

A retrospective cross-sectional study.

**Setting:**

All children with foreign body aspiration at Fujian Maternity and Child Health Hospital, Affiliated Hospital of Fujian Medical University from January 2015 to December 2020.

**Patients:**

Children with FB removal using a flexible bronchoscopy were enrolled in the trial according to the inclusion criteria.

**Measurements:**

Both multivariable and logistic regression analyses were used to analyze the association between characteristic data and reintubations. The results were presented as odds ratios (ORs) with 95% confidence intervals (CIs).

**Results:**

In total, 244 patients met with the inclusion criteria and were included in the analysis. Among those participants, 28 children (11.5%) underwent reintubations after FB removal by flexible bronchoscopy. Independent factors associated with reintubations were identified as operative time ≥ 60 min [OR: 3.68, 95% CI (1.64–8.82)] and ASA ≥ III [OR: 5.7, 95% CI (1.23–26.4)].

**Conclusions:**

Children undergoing FB removal by a flexible bronchoscopy may encounter with a high incidence of postoperative reintubations. Both long operative duration and a severe physical status cause a growing risk of reintubations.

## Introduction

Foreign body (FB) aspiration usually results in an asphyxiation and even accidental death in children younger than 4 years [1]. The morbidity ranges from 0.3 to 23% in children undergoing FB removal by a bronchoscopy [1–3]. Flexible bronchoscopy is becoming a more popular than rigid bronchoscopy to remove FBs [4, 5]. Reintubation is a severe complication occurring in both flexible bronchoscopy and rigid bronchoscopy [6]. Reintubations lead to prolonged mechanical ventilator support, increased postoperative ICU admission, and a higher cost [6]. Identifying and focusing on children with higher risks of reintubations are crucial.

A large scale of investigation has reported that there’re risks correlated with reintubations, including age younger than 1 year, patients with a higher American Society of Anesthesiology (ASA) physical status, and a longer operative duration [7]. Age less than 1 year is proved to be an independent risk factor for reintubations in the early postoperative stage [8]. A long operative duration is revealed to be a greater risk factor for intubations, ranging from 180 min to more than 6 h [8–10]. However, the risk factors for reintubations in children with FB removal using a flexible bronchoscopy remain unclear. The mean age of children with FB removal was reported as 3.7 years [8], the mean operative duration was published to be 27.4 min [2]. Thus, whether a younger age and a longer procedure time are higher risk factors for FB removal must be determined. Both a younger age and a longer operative duration likely cause tissue edema. In addition, different FB types may lead to distinctive airway tissue trauma or inflammation.

We hypothesized that risk factors for reintubations in children with FB removal were similar to those of reintubations with an early postoperative duration, including age and operative duration. The study was designed to determine the risk factors for reintubations with FB removal among children. Thus, we reviewed patients who had undergone FB removal surgery at our hospital in the last six years, in order to perform a retrospectively analyze eligible cases with reported clinical factors.

## Methods

### Study design and data resources

 The study was designed as a single-center retrospective cross-sectional study and was approved by the Research Committee of Fujian Maternity and Child Health Hospital, Affiliated Hospital of Fujian Medical University (2021KLR09069). Participants were longitudinally surveyed from the anesthesia information management system in our hospital from January 1, 2015 to December 31, 2020.

The inclusion criteria were children who had undergone FB removal using a flexible bronchoscope under general anesthesia with LMA (LTLAN; Funia Medical Equipment Co., Ltd. Zhuhai, China). The exclusion criteria were as follows: children with preoperative mechanical ventilation and children who had undergone bronchoscopy procedures twice during hospital residency.

### Procedures

**Fig. 1:**
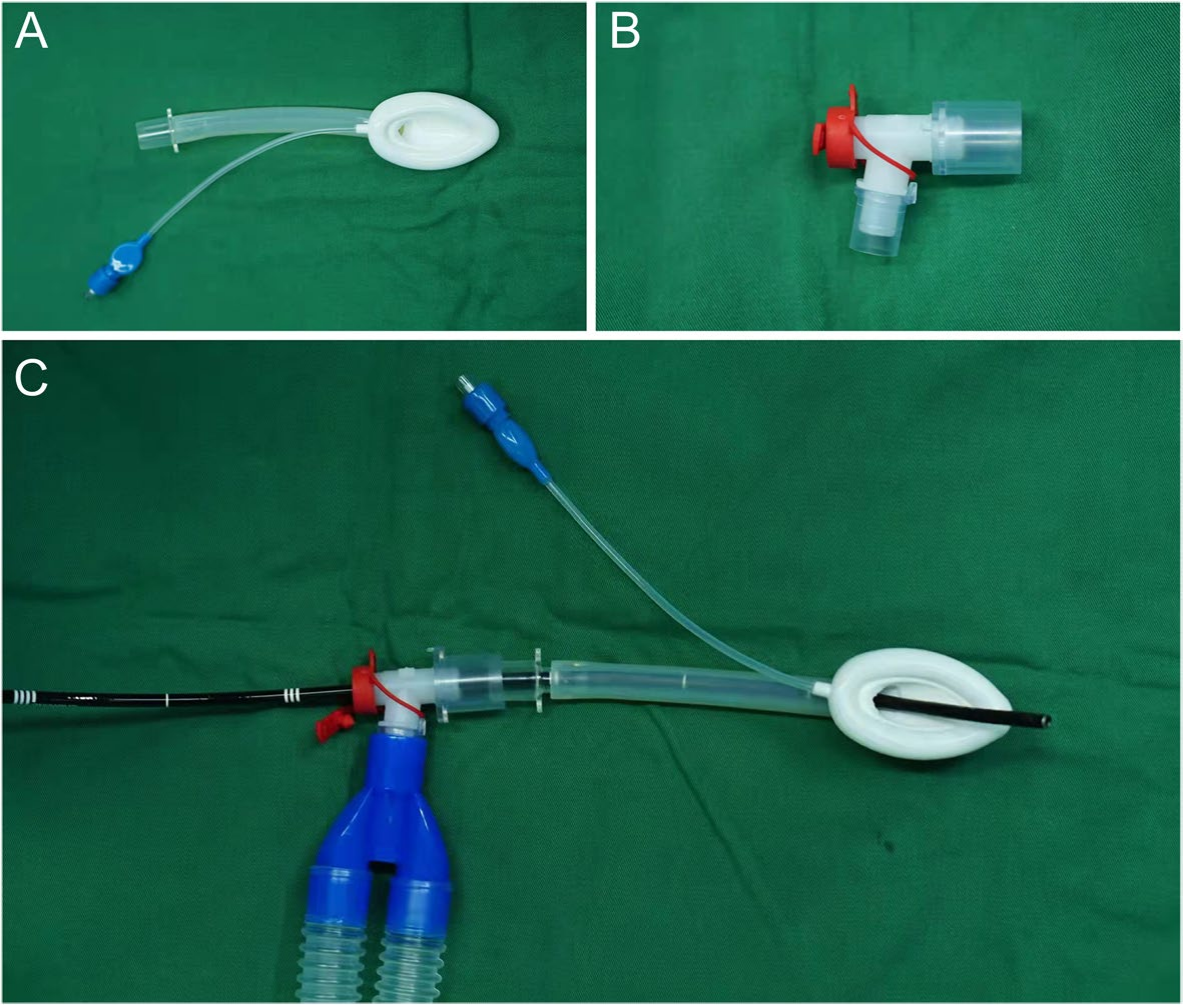
Adjusted laryngeal mask airway (LMA) combined with flexible bronchoscopy. **A** The distal apertures of the LMA were excised using medical scissors. The LMA size was decided by the weight of participants. **B** The swivel connector. **C** Flexible bronchoscopy was inserted through both the swivel connector and LMA

Before anesthesia initiation, all the children were injected with atropine (0.01 mg/kg), dexamethasone (0.2 mg/kg), and 1% lidocaine (1.5 mg/kg) by venous injection. Anesthesia was induced by propofol (3–5 mg/kg), remifentanil (2 ug/kg), along with rocuronium (0.6 mg/kg) or mivacurium (0.2 mg/kg) or without muscle relaxants. After the induction, an adjusted LMA (the size of the mask depending on the child’s weight) (Fig. 1A) was inserted by hand. A flexible pediatric bronchoscope (Olympus flexible bronchoscope BF-P 290, Japan) was passed through the swivel connector (Fig. 1B), which was connected the LMA and anesthesia equipment (Fig. 1C). Grasping forceps was used to remove FB through a flexible bronchoscope. Anesthesia was maintained by the infusion of propofol (100–150 µg/kg/min) and remifentanil (0.3–0.4 µg/kg/min), with bolus doses of rocuronium (0.3 mg/kg) or mivacurium (0.1 mg/Kg) in accordance with clinical indications. Mechanical ventilation was set as the pressure control mode to control respiration during the procedure, and the parameters of respirator were adjusted based on the actual oxygen situation (SpO_2_) and the end-tidal carbon dioxide (PetCO_2_).

### Clinical characteristics and outcome collection

Clinical documents were collected, such as characteristics regarding the patient (age, gender, and weight), FB (duration, type, size, and location), operation (operative time, intraoperative airway hemorrhage), and anesthesia (neuromuscular blockade agents and ASA classification). Additionally, other crucial information was also recorded, such as radiological findings (pneumonia, pulmonary emphysema, and atelectasis), perioperative adverse events including hypoxemia (pulse oxygen saturation below 90%), laryngospasm (stridor on inspiration with closure of vocal cord closure), pneumothorax, glottic edema, bronchial laceration, and reintubation.

The main outcome was reintubation. Reintubation was defined as the unplanned placement of the endotracheal tube after LMA removal not as a mid-procedure. Hypoxemia after LMA removal was referred to as postoperative pulse oximetric saturation (SpO_2_)/fraction of inspired oxygen (FiO2) ratio falling below 201 [11]. Reintubation was performed if one child failed to reverse hypoxemia with a mask-manipulated ventilation supported by pure oxygen, who encountered with a severe glottic edema, pneumothorax, pneumorrhagia or other life-threatening complications. Reintubation was determined by a senior anesthesiology, evaluating the necessity and benefit of the performance.

Several variables were considered subvariables, such as age in years (divided into <1, 1–2, 2–3, ≥ 3), weight in kilograms (kg) (divided into < 10, 10–20, ≥ 20), duration of FB in terms of days (divided into < 5, 6–30, > 30), type of FB, location of FB, maximum dimension in FB according to millimeters (mm) (divided into < 5, 5–10, 10–15, ≥ 15), operative time in minutes (divided into < 60, ≥ 60), ASA classification, neuromuscular blockade agents, the development of airway hemorrhage, and radiological findings (pneumonia, emphysema, and atelectasis).

### Statistical analyses

Selected characteristics was shown to be frequency distribution (categorical variable) in Table 1. As our primary analysis, we defined cases as those who accepted reintubations after FB removal. Multivariate logistic regression included sex, age, weight, FB information (including location, type, duration, and maximum dimension), ASA rank, muscle relaxant, operative time, airway hemorrhage, and radio findings. Cases or controls with preoperative ventilation or missing data were excluded. The enumeration data were documented as numbers or percentages. Pearson’s chi-squared test was used to analyze comparisons between subvariables. All the variables with a *P* value ≤ 0.2 in the single-factor analysis were considered for further multivariate analysis using a logistic regression. Odds ratios (ORs) with 95% confidence intervals (CIs) were used to express results of the regression analysis data. Statistical significance was defined as a *P* value < 0.05. All the data were analyzed by IBM Corp. Released 2015. IBM SPSS Statistics for Windows, Version 23.0 (IBM Corp., Armonk, NY, USA).

**Table 1:** The characteristics of all children and risk factors associated with reintubation

Variables	Number (*n* = 244)	RE (*n* = 28)	URE (*n* = 216)	OR and 95%CI	*P* value
Gender					
Female	80 (32.7%)	11	69	1.38 (0.61–3.10)	0.436
Male	164 (67.2%)	17	147	1	-
Age (year)					
< 1	12 (4.9%)	1	11	0.58 (0.06–5.31)	1.000
1–2	173 (70.9%)	21	152	0.87 (0.33–2.31)	0.788
2–3	44 (18.0%)	6	38	1	-
≥ 3	15 (6.2%)	0	15	-	-
Weigh (kg)					
< 10	57 (23.4%)	4	53	0.49 (0.16–1.49)	0.202
10–20	181 (74.2%)	24	157	1	-
≥ 20	6 (2.5%)	0	6	-	-
FB location					
Trachea	13 (5.3%)	3	10	1	-
Right bronchus	118 (48.4%)	11	107	0.34 (0.08–1.43)	0.146
Left bronchus	111 (45.5%)	14	97	0.48 (0.12–1.96)	0.386
Multiple sites	2 (0.8%)	0	2	-	-
FB type					
Organic	214 (87.7%)	26	188	1.94 (0.44–8.61)	0.545
Inorganic	30 (12.3%)	2	28	1	-
FB max dimension (mm)					
< 5	56 (23.0%)	8	48	1	-
5–10	122 (50.0%)	11	111	0.60 (0.22–1.57)	0.290
10–15	55 (22.5%)	6	49	0.73 (0.24–2.28)	0.592
≥ 15	11 (4.5%)	3	8	2.25 (0.49–10.32)	0.371
FB duration (days)					
≤ 5	148 (60.7%)	11	137	1	-
6–30	76 (31.1%)	12	64	2.34 (0.98–5.58)	0.051
> 30	20 (8.2%)	5	15	4.15 (1.27–13.56)	0.026
ASA					
< III	232 (95.1%)	21	211	1	
≥III	12 (4.9%)	5	7	7.18 (2.09–24.6)	0.004
Muscle relaxant					
No	20 (8.2%)	2	18	1	-
Mivacurium	84 (34.4%)	6	78	0.69 (1.13–3.72)	0.648
Rocuronium	140 (57.4%)	20	120	1.50 (0.32–6.97)	1.000
Operative time(mins)					
< 60	190 (77.9%)	14	176	1	
≥ 60	54 (22.1%)	14	20 (40)	8.8 (3.68–21.08)	0
Airway hemorrhage	56 (23.0%)	13	43	3.02 (1.33–6.85)	0.006
Radiological findings					
Pneumonia	115 (47.1%)	10	105	2.59 (0.26–1.33)	0.198
Emphysema	101 (41.4%)	13	88	1.25 (0.57–2.76)	0.579
Atelectasis	6 (2.5%)	2	4	4.06 (0.71–23.25)	0.090

## Results

**Fig. 2:**
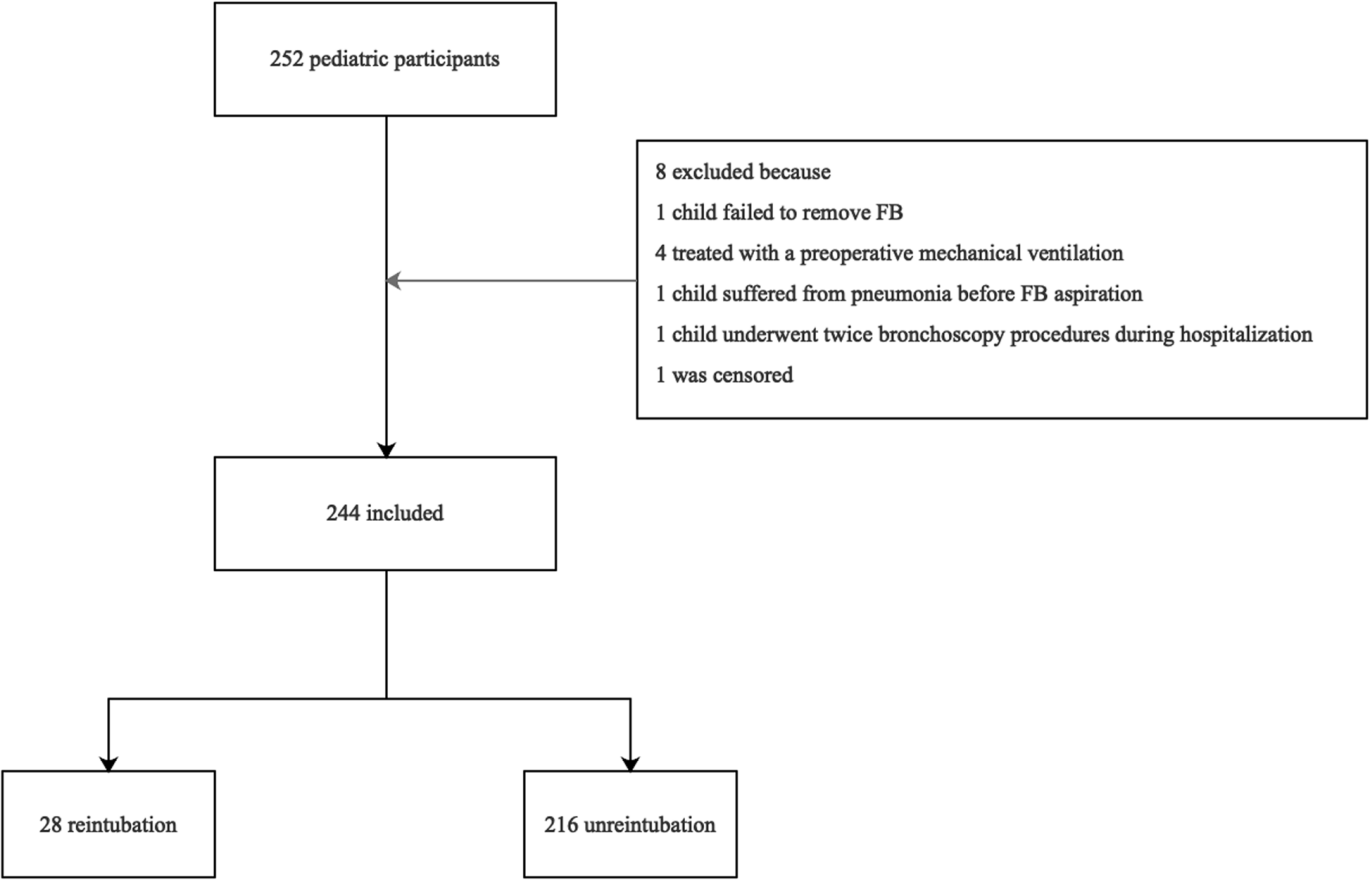
Flow chart of the participants, events, and inclusion analyses

Of 252 children who had undergone FB removal using a flexible bronchoscopy between 2015 and 2020, 8 participants were excluded because of FB removal failure, a preoperative mechanical ventilation, pneumonia before FB aspiration, two bronchoscopy procedures during hospitalization, and censored data. All of 244 patients were included in the analysis. Among the participants, 28 children had undergone reintubations (11.5%) (Fig. 2). The characteristics of all the patients are summarized in Table 1. The ages of the patients ranged from 9 months to 11 years, and most of the children (93.8%) were younger than 3 years. A total of 164 (67.2%) patients were male. FB aspiration was observed in the right bronchus of 118 children (48.4%), the left bronchus of 111 (45.5%) children, the trachea of 13 (5.3%) children, and more than one site in 2 (0.8%) children. Most of the FBs (87.7%) were organic and 12.3% were inorganic. More than half of the children (60.6%) had undergone intervention within 5 days of the aspiration event or onset of symptoms, 76 children (31.2%) had an intervention between 6 and 30 days and 20 (8.2%) children had been treated for more than 30 days. Half of the children inhaled FBs with a max dimension from 5 to 10 mm, 56 (23.0%) children with < 5 mm, 54 (22.1%) children from 10 to 15 mm, and the remainder had the max dimension of > 15 mm. Except 1 child with an X-ray, all the participants were scanned to identify aspiration of FB using CT. Thus, 4 children were excluded without FB aspiration. The mean operative time was 47.3 min, and 22.1% of patients had a mean operative time > 60 min. Flexible bronchoscopy was conducted without any muscle relaxants in 20 (8.2%) children, with mivacurium in 84 (34.4%) children, with rocuronium in 140 (57.4%) children. Hypoxemia was observed in 38 (15.6%) children, laryngospasm in 54 (22.1%) children, and glottic edema in 71 (29.1%) children.

### Risk factors associated with reintubation

Risk factors associated with reintubation (with *P* value ≤ 0.2) were FB in the right bronchus [OR 0.34, 95% CI (0.08–1.43)], an FB duration of 6–30 days [OR: 2.34; 95% CI (0.98–5.58)], an FB duration > 30 days [OR: 4.15; 95% CI (1.27–13.56)], ASA ≥ III [OR: 7.18; 95% CI (2.09–24.6)], pneumonia [OR: 2.59; 95% CI (0.26–1.33)], atelectasis [OR: 4.06; 95% CI (0.71–23.25)], an operative duration ≥ 60 min [OR: 8.8; 95% CI (3.68–21.08)], and intraoperative emphysema [OR; 3.02; 95% CI, 1.33–6.85)] (Table 1).

### Multivariate logistic regression analysis

Independent predictors of reintubations were an operative time of ≥ 60 min [OR: 3.8; 95% CI (1.64–8.82)] and ASA ≥ III (OR; 5.70; 95% CI, 1.23–26.40)], in accordance with the results of logistic regression analysis (Table 2).

**Table 2:** Multivariate analysis of factors associated with reintubation

Variables	OR & 95%CI	*P* value
ASA not less than III	5.70 (1.23–26.4)	0.026
Operative duration (≥ 60 min)	3.68 (1.64–8.82)	0.002
FB location	0.80 (0.39–1.61)	0.296
FB duration (days)	1.01 (0.66–1.54)	0.97
Radiological findings		
Pneumonia	1.00 (0.42–2.39)	0.998
Atelectasis	4.75 (0.77–29.17)	0.096
Airway hemorrhage	1.71 (0.67–4.37)	0.321

## Discussion

In the present study, we revealed that a longer operative duration (not less than 60 min) and ASA ≥ III are independent predictors of reintubations in children undergoing FB removal using a flexible bronchoscopy. These findings suggested that it is essential to take preventive and pretreatment measures must be adopted to manage reintubations occurring in this subgroup of children, like intrabronchial epinephrine.

Reintubation is regarded as a severe complication of numerous surgical procedures. Regardless of adhering to the authorized extubation criteria of extubate, we retrospectively observed 28 (11.5%) children undergoing reintubations, a number higher than that associated with general surgery (0.14-6.9%) [10, 12, 13]. Consistent with previous studies, we identified several independent risk factors implicated in reintubations, such as a long operative duration and ASA ≥ III [8]. Unlike general surgery, other risk factors were associated with reintubations after FB removal in this trial, including an age < 1 year, emergency cases, and neuromuscular blocking agents [8]. In line with the epidemiologic and demographic data from published reports [12, 14], children aged from 1 to 3 years accounted for 86.1% (217 cases) of all the participants, indicating that an age younger than 1 year is a risk factor. An age less than 1 year is a risk factor for reintubations, because of the anatomical and physical characteristics, lower functional reserve capacity, and higher oxygen consumption [15]. Thus, caution must be applied to younger (1 year) children undergoing FB removal in the clinical practice.

Both an operative time > 3 h and surgeon experience are well-established risk factors for reintubation during early general postoperative procedures [8]. An operative time ≥ 60 min, but not > 3 h, was identified as a risk factor for reintubation [12]. The difference can be explained by children with FB being usually complicated with pulmonary complications and the deteriorative trachea and glottis resulting from the procedure of flexible bronchoscopy. In the present investigation, FBs were extracted primarily by fibrotic bronchoscopy under general anesthesia using a first-generation laryngeal mask, eliciting an operative time of most cases less than 60 min in most cases. Despite rigid bronchoscopy using a high frequency jet ventilator was considered the gold standard for FB removal, being flexibility, visibility, and little trauma contributed to the widespread usage of flexible bronchoscopy for FB extraction [16]. Additionally, flexible bronchoscopy improved the success rate ranging from 75 to 100% [12]. This finding renders it suitable to shorten the operative time of FB removal, in addition to its greater rate.

Most of the FBs (87.7%) were identified as organic materials, particularly nuts (e.g., peanuts) and seeds (e.g., sunflower and watermelon seeds). Organic materials can absorb fluid and swell, and the oils from nuts can cause localized inflammation and mechanical obstruction [17]. However, our findings are insufficient to support the correlation between organic FBs and increased reintubation, similar to the study reported by Charlotte K [18]. Similar to the FB duration, the FB size is not a risk factor for FB removal, a finding that may seem unreasonable. Previous studies have proposed that a long FB duration may cause airway edema, granulation tissue, and infection [19]. In addition to the FB, cylindrical and spherical items can occlude the airway or tend to slip as they cross the glottic chink, prolonging the operative time [19]. In contrast to an ASA greater than III, available data have demonstrated that there is no significant difference among preoperative complications including pneumonia, airway hemorrhage, and atelectasis. We believe that a large sample size and a prospective controlled trial will reveal the relationship among these susceptibility factors and reintubation.

The present study has several limitations. First, our data cannot avoid some bias because the study was designed as a retrospective, single-center, cross-sectional investigation. Second, it seems likely to incorrectly eliminate actual risk factors, particularly severe glottic edema, and severe airway spasms. Third, some confounding factors are difficult to be ruled out, including the growth in the experience of anesthesiologists and surgeons. Finally, children with preoperative mechanical ventilation were removed before decreasing the relationship between ASA and reintubation.

## Conclusions

Taken together, children undergoing FB removal by a flexible bronchoscopy have a higher risk of postoperative reintubations. Our findings suggested that children are susceptible to reintubations given both long operative duration and ASA ≥ III.

## Data Availability

The datasets used and/or analysed during the current study available from the corresponding author on reasonable request.
